# Change in alcohol and tobacco consumption after a diagnosis of head and neck cancer: Findings from Head and Neck 5000

**DOI:** 10.1002/hed.25116

**Published:** 2018-02-27

**Authors:** Chris M. Penfold, Steven J. Thomas, Andrea Waylen, Andrew R. Ness

**Affiliations:** ^1^ National Institute for Health Research Bristol Biomedical Research Centre University Hospitals Bristol NHS Foundation Trust and University of Bristol Bristol UK; ^2^ School of Oral and Dental Sciences University of Bristol Bristol UK

**Keywords:** alcohol, head and neck cancer, health behaviors, smoking, teachable moment

## Abstract

**Background:**

Tobacco and alcohol consumption are risk factors for developing head and neck cancer, and continuation postdiagnosis can adversely affect prognosis. We explored changes to these behaviors after a head and neck cancer diagnosis.

**Methods:**

Demographic and clinical data were collected from 973 people newly diagnosed with oral cavity, oropharyngeal, or laryngeal cancer. Tobacco and alcohol consumption were additionally collected 4 and 12 months later.

**Results:**

The prevalence of high alcohol consumption reduced from 54.3% at diagnosis to 41.4% at 12 months, and smoking reduced from 21.0% to 11.7%. Changes in behavior were dynamic, for example, 44% of smokers at 12 months were not smoking at diagnosis or 4 months. Several factors were associated with alcohol consumption, whereas only tumor site and comorbidities were associated with smoking.

**Conclusion:**

A diagnosis of head and neck cancer can result in important changes in alcohol consumption and smoking prevalence. However, these changes are dynamic in the first year after diagnosis.

## 1 INTRODUCTION

Head and neck cancer is an important cause of mortality and morbidity worldwide.^1^, ^2^ In the United Kingdom, survival rates for some head and neck cancers have improved in the last 25 years.^3^, ^4^ The reasons for these improvements are unclear but could include alterations in lifestyle behavior after treatment.

Tobacco and alcohol consumption are established risk factors for developing head and neck cancer.^5^, ^6^, ^7^, ^8^ Continued tobacco and alcohol consumption after treatment for head and neck cancer is a risk factor for the development of second primary cancers,^9^, ^10^ and decreased 5‐year survival rates.^11^ Tobacco consumption during treatment is also associated with poorer outcomes of surgical^12^, ^13^ and nonsurgical treatments.^11^, ^14^, ^15^, ^16^ Tobacco and alcohol consumption are, therefore, potentially important modifiable factors that may influence disease recurrence and survival.

A diagnosis of head and neck cancer may be a “teachable moment,” which leads individuals to spontaneously adopt risk‐reducing health behaviors.^17^ One study suggested that approximately 50% of people who were smokers or problem drinkers when diagnosed with head and neck cancer had quit smoking or reduced their alcohol consumption to within safe limits 12 months after diagnosis with no specific health behavior advice above usual care.^18^ Smoking and heavy alcohol consumption are interrelated,^19^ heavy alcohol drinkers are less likely to try to stop smoking, and are less successful if they try.^20^, ^21^, ^22^

Previous studies, which included prediagnosis and postdiagnosis measures of smoking and/or alcohol consumption after a diagnosis of head and neck cancer, had relatively small sample sizes (n < 300).^18^, ^23^, ^24^, ^25^, ^26^ Repeated measures of smoking status postdiagnosis were included in some studies,^18^, ^25^, ^26^ but none had repeated measures of postdiagnosis alcohol consumption. Using data from a large, prospective clinical cohort, we explored whether people changed their alcohol and tobacco consumption between diagnosis for head and neck cancer and 12 months later, and whether these changes were stable during this period. We also explored which factors were associated with these changes.

## 2 MATERIALS AND METHODS

Data were collected from participants in the Head and Neck 5000 prospective clinical cohort study. Details on Head and Neck 5000 have been published previously,^27^, ^28^ and a fully searchable data dictionary is available online (http://www.headandneck5000.org.uk/). In brief, the Head and Neck 5000 aimed to recruit 5000 adults newly diagnosed with head and neck cancer between April 2011 and December 2014 from 76 UK centers. People with lymphoma, skin tumors, or a recurrence of a previous head and neck cancer were excluded.^27^ The study was approved by the National Research Ethics Committee (South West Frenchay Ethics Committee, reference 10/H0107/57, November 5, 2010) and approved by the research and development departments for participating National Health Service trusts. We collected data at diagnosis (baseline), and 4 and 12 months after using self‐report questionnaires and data capture forms to record details from clinical records. Five thousand five hundred eleven people consented into the study, of whom 138 were subsequently found to be ineligible. The resultant study sample contained 5369 people.

### 2.1 Inclusion criteria

For this study, we included participants who returned a baseline “About You” questionnaire, and who had oral cavity, oropharyngeal, or laryngeal cancer. We excluded people on a palliative or supportive treatment pathway at diagnosis. This was a relatively small group and we expected them to have different motivations to change their health behaviors after diagnosis than people on a curative treatment pathway. We also excluded people who would not have been able to complete data at all 3 time points, due to having died before the 12‐month data collection or who declined to complete any or all of the baseline, 4‐month, or 12‐month questionnaires.

### 2.2 Measures

#### 2.2.1 Health behaviors: Alcohol and tobacco consumption

Consumption of alcohol and tobacco were recorded through self‐report questionnaires distributed at diagnosis (baseline), and 4 and 12 months after diagnosis. People who responded “none” when asked “In a typical week, how many days do you drink alcohol?” were assumed to consume no units of alcohol per week. The amount and frequency of consumption per week of beer, spirits, or wine were combined and converted into standard UK alcohol units per week using the method described by Zuccolo et al.^29^ We categorized participants' alcohol consumption using the revised UK Department of Health guidelines^30^ and included additional higher thresholds defined by the “Institute of Alcohol Studies”^31^ as well as a category for people who did not consume alcohol. This resulted in 4 categories of alcohol consumption: low consumer: (1) nondrinker: 0 units/week; (2) moderate use: > 0 and ≤14 units/week; high consumer: (3) harmful use: women > 14 and ≤35 units/week, men > 14 and ≤50 units/week; and (4) hazardous use: women > 35 units/week, men > 50 units/week.

Tobacco consumption at diagnosis was recorded as “current user of tobacco,” “former user of tobacco,” or “never used tobacco.” In the 4‐month and 12‐month questionnaires, tobacco consumption was recorded as “current user” or “recently quit using tobacco or never used tobacco,” which were recategorized as “current smoker” and “current nonsmoker,” respectively.

### 2.3 Demographic factors

We included the following demographic factors reported in previous studies to be associated with change in smoking or alcohol behavior: age, sex, marital status, and education. The participants' sex and marital status were recorded in the baseline “Health and Lifestyle” questionnaire. Marital status was dichotomized into “married/living with partner” and “single, divorced, widowed, or separated.” Participants' age was derived from their date of birth and date of consent.

### 2.4 Clinical data

Tumor site,^32^ disease stage,^32^ and the type of treatment received^23^, ^25^ have previously been found to be associated with change in smoking or alcohol behavior and have, therefore, been included as potential factors thought to be associated with behavior change in our study. We also included prediagnosis comorbidity. Tumor site was defined using the categorized International Classification of Disease‐10 codes for tumor location, which have been described previously.^28^ Pretreatment clinical TNM classification was categorized into “early stage” (stages I and II) and “advanced stage” (stages III and IV). Pretreatment comorbidity, using the overall comorbidity score from the Adult Comorbidity Evaluation‐27,^33^ was categorized into “no comorbidity, mild decompensation, or unknown” and “moderate or severe decompensation.” The actual treatment the participants received was recorded in the 4‐month data capture form. We grouped these treatments into 4 categories: (1) surgery only; (2) surgery + adjunct therapy; (3) combined chemoradiotherapy; and (4) radiotherapy only.

### 2.5 Study samples

Some participants had missing data, either from nonresponse to individual questions or from attrition of the cohort over time due to death or loss to follow‐up. The proportion of “Health and Lifestyle” questionnaires returned at diagnosis was 74.9%, 73.0% at 4 months, and 58.6% at 12 months (excluding people who died by 4 and 12 months, respectively). Five people were marked as “lost to follow‐up” at 12 months for questionnaire data. We defined our main study sample as those participants with complete data for exposures and outcomes at all time points.

### 2.6 Statistical analysis

In order to determine whether people in the main study sample differed from people who met the inclusion criteria but had some missing data, we compared the demographic and clinical characteristics of these groups. Continuous measures were compared using *t* tests and categorical measures compared using chi‐square tests. Health behaviors at each time point were described using proportions. We described the change in health behaviors from diagnosis to 4 months and diagnosis to 12 months. We also described behavior trajectories using all 3 time points to identify whether behaviors changed during the 12 months and if change occurred early (by 4 months), late (4‐12 months), or was only temporary.

In order to explore associations between proposed risk factors and change in health behaviors, we stratified analyses by prediagnosis health behavior. For smoking, we further divided baseline nonsmokers into “never” and “former” smokers. For each stratum of prediagnosis health behaviors, we used binary logistic regression models to calculate unadjusted odds ratios (ORs) of being in the “unhealthy” behavior category (being a high consumer of alcohol or current smoker) as opposed to being in the “healthy” category (low consumer of alcohol or not current nonsmoker) at 4 months and at 12 months for each of the proposed risk factors. We then included risk factors in mutually adjusted binary logistic regression models. All analyses were undertaken in Stata 14 (StataCorp, 2015).

### 2.7 Sensitivity analyses

We repeated our cross‐sectional analyses of health behaviors at each of the time points separately using those respondents with complete data for exposures and outcomes at that time point. In order to determine whether recent changes in recommended alcohol consumption for men affected our findings, we repeated our analyses of alcohol consumption at 4 months and 12 months using the previous guideline for maximum alcohol consumption for men of ≤21 units/week.

## 3 RESULTS

### 3.1 Description of study samples

**Figure 1:**
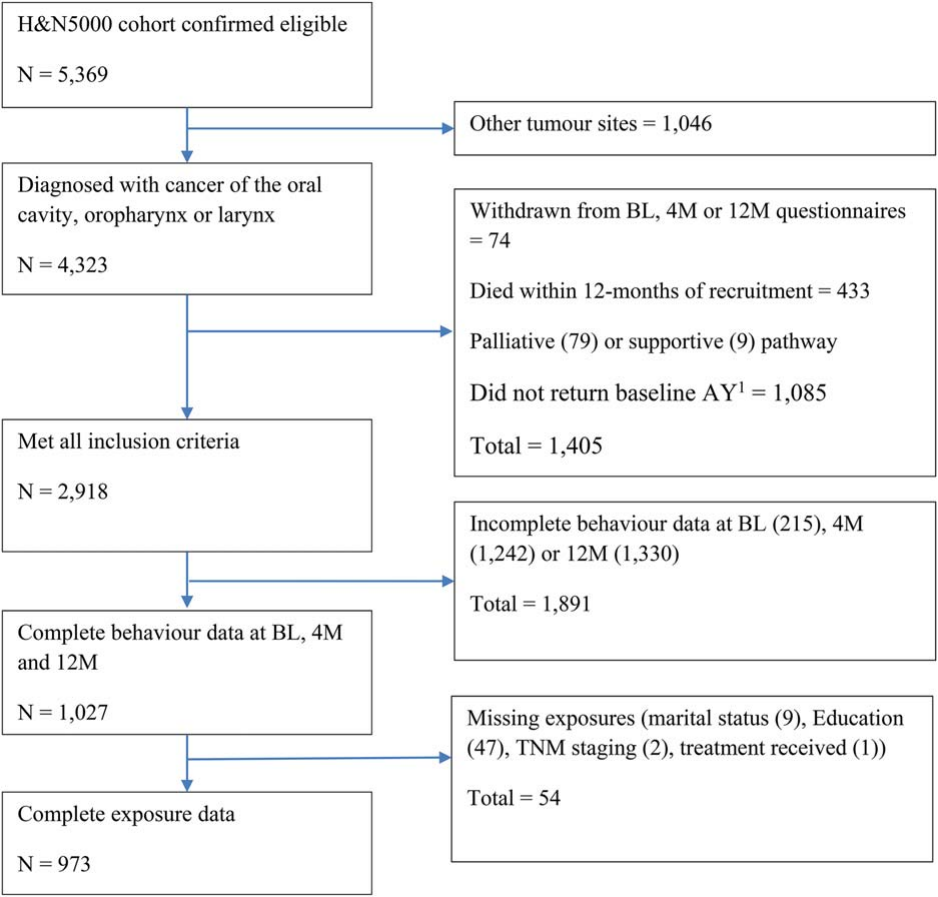
Flow diagram of the inclusion and exclusion of eligible participants from the Head and Neck 5000 (H&N5000) clinical cohort. 4M, 4 month; 12M, 12 month; AY, About You questionnaire, BL, baseline [Color figure can be viewed at http://wileyonlinelibrary.com]

From the total Head and Neck 5000 cohort who were confirmed as eligible to participate (N = 5373), 4326 people (80.5%) were diagnosed with oral cavity, oropharyngeal, or laryngeal cancer and 2916 (67.4%) of these met the inclusion and exclusion criteria (see Figure 1). Nine hundred seventy‐three (33.4%) of these people had complete data for potential exposures and health behaviors at diagnosis, 4 months, and 12 months. People with complete data were slightly older, and more likely to be men, have higher levels of education, and have early‐stage disease than those who met the inclusion criteria but were missing data (Supporting Information Table S1). The weekly alcohol consumption of people with complete data was higher at all time points, whereas tobacco consumption only differed at diagnosis, when people in the main study sample were more likely to be current smokers and less likely to be never smokers.

### 3.2 Health behaviors at diagnosis, 4 months, and 12 months

#### 3.2.1 Alcohol

**Figure 2:**
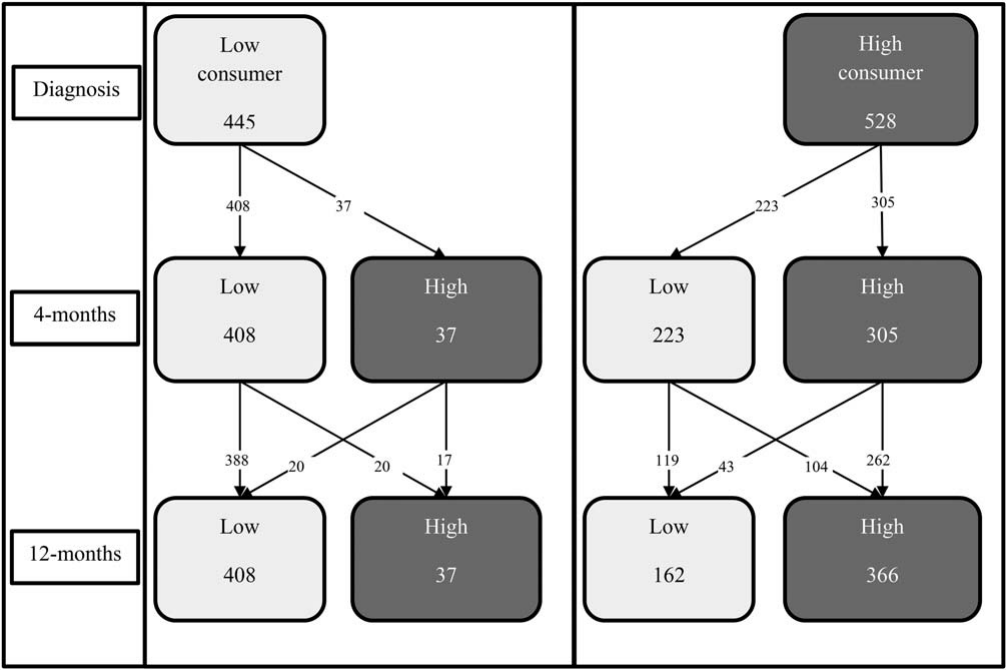
Trajectories of alcohol consumption at diagnosis, 4 months, and 12 months

At diagnosis, 54.3% of people (N = 528) in the main sample consumed more than the recommended weekly maximum number of units of alcohol and were considered high consumers (Table 1), reducing to 35.2% (N = 342) at 4 months and increasing slightly to 41.4% (N = 403) at 12 months. The majority of high alcohol consumers at 4 months were high consumers at diagnosis (89.2%; 305 of 342), but a third of low consumers at 4 months were high consumers at diagnosis (35.3%; 223 of 631; see Figure 2). Few high consumers at 12 months were low consumers at diagnosis (9.2%; 37 of 403), but nearly one‐third (30.8%; 124 of 403) of high consumers at 12 months had changed from being low consumers at 4 months.

**Table 1:** Categories of alcohol and tobacco consumption at diagnosis, 4 months, and 12‐months

	Diagnosis no. of people (%)	4 mo no. of people (%)	12 mo no. of people (%)
Grouped weekly alcohol consumption			
“Healthy” drinker (≤14 units/wk)	445 (45.7)	631 (64.9)	570 (58.6)
Nondrinker	242 (24.9)	415 (42.7)	320 (32.9)
Moderate use	203 (20.9)	216 (22.2)	250 (25.7)
“Unhealthy” drinker (>14 units/wk)	528 (54.3)	342 (35.2)	403 (41.4)
Hazardous use	367 (37.7)	272 (28.0)	327 (33.6)
Harmful use	161 (16.6)	70 (7.2)	76 (7.8)
Tobacco consumption			
Current smoker	204 (21.0)	105 (10.8)	114 (11.7)
Former^a^	564 (58.0)	868 (89.2)	859 (88.3)
Never^a^	205 (21.1)		

a “Former” and “Never” smokers are combined at the 4‐month and 12‐month questionnaires.

#### 3.2.2 Smoking

**Figure 3:**
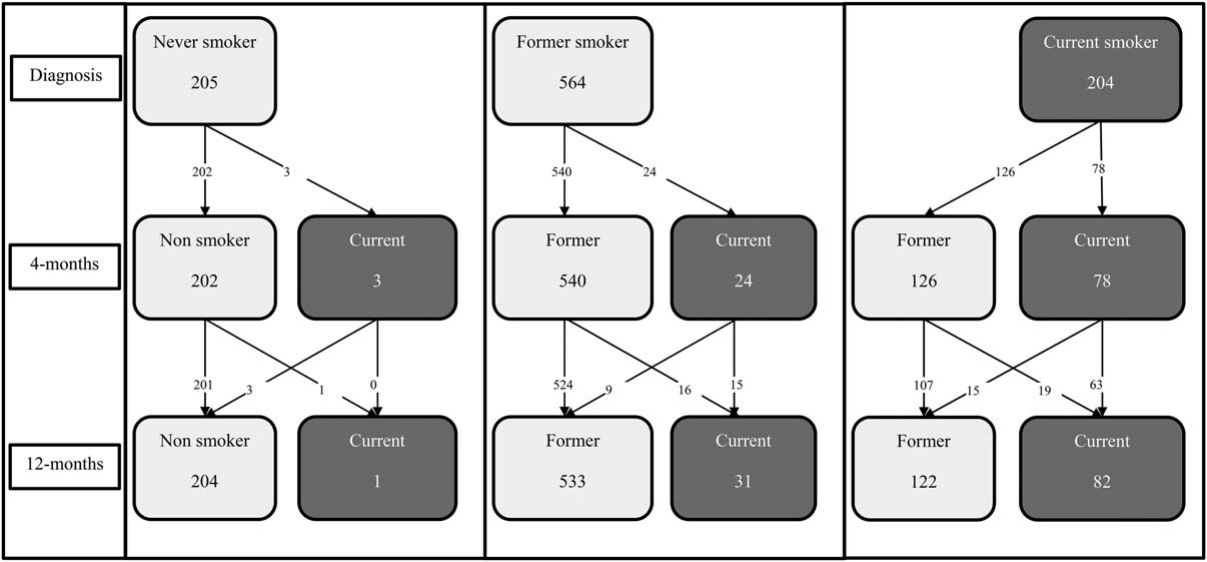
Trajectories of smoking behavior at diagnosis, 4 months, and 12 months

At diagnosis, 21.0% of participants (N = 204 of 973) in the main sample were current smokers, 58.0% (N = 564) were former smokers, and 21.1% (N = 205) had never smoked (Table 1). Four months after diagnosis, smoking prevalence had halved to 10.8% (N = 105), and remained at this level at 12 months (11.7%; N = 114). The 205 people who had never smoked at diagnosis remained nonsmokers at 4 months (98.5%) and 12 months (99.5%), and so have been excluded from descriptions of change in behavior and further analyses. A quarter of the smokers at 4 months were former smokers at diagnosis (23.5%; 24 of 102; see Figure 3). Half of the current smokers at 12 months were smokers at both diagnosis and 4 months (56%; 63 of 113). A third of the current smokers at 12 months were former smokers at 4 months (31.0%; 35 of 113), of whom nearly half (46%; 16 of 35) were former smokers at diagnosis. A comparable number but much smaller proportion (3.7%; 24 of 655) of former smokers at 12 months were current smokers at 4 months.

### 3.3 Factors associated with postdiagnosis behavior change

#### 3.3.1 Logistic regression models for health behaviors at 12 months

Health behavior at 12 months was the main outcome of our logistic regression models (Tables 2 and 3), with results for 4 months included briefly in the text here and in detail in the Supporting Information Tables S2 and S3). In summary, the adjusted models found that being men, having a laryngeal tumor, and being a former or current smoker at diagnosis were associated with high alcohol consumption at 12 months. People with oropharyngeal tumors were less likely to restart smoking, whereas continuing to smoke was associated with being men, having an oral cavity or oropharyngeal tumor, and having more severe comorbidities at diagnosis.

**Table 2:** Unadjusted and mutually adjusted logistic regression models for odds of consuming alcohol above the recommended limits at 12 months postdiagnosis, stratified by prediagnosis alcohol consumption

	Low consumers						High consumers					
	Unadjusted			Mutually adjusted^a^			Unadjusted			Mutually adjusted^a^		
	OR	95% CI	*P* value	OR	95% CI	*P* value	OR	95% CI	*P* value	OR	95% CI	*P* value
Age, ref < 55 y												
55‐69	0.82	0.36‐1.85	.63	0.66	0.28‐1.61	.36	1.20	0.76‐1.89	.44	1.10	0.67‐1.82	.71
70+	0.86	0.34‐2.15	.74	0.56	0.20‐1.61	.28	1.51	0.80‐2.84	.20	1.24	0.61‐2.50	.56
Sex, ref male												
Female	0.46	0.20‐1.08	.07	0.48	0.19‐1.20	.12	0.41	0.26‐0.66	< .001	0.36	0.21‐0.59	< .001
Marital status, ref married/cohabiting												
Single, divorced, widowed, or separated	0.82	0.39‐1.74	.61	0.83	0.37‐1.87	.65	1.14	0.77‐1.71	.51	0.96	0.61‐1.50	.85
Education, ref school level												
Further education	1.47	0.69‐3.11	.31	1.76	0.78‐3.95	.17	1.27	0.84‐1.93	.26	1.39	0.88‐2.18	.15
University/poly	1.10	0.43‐2.81	.84	1.49	0.51‐4.33	.46	1.08	0.66‐1.78	.75	1.38	0.80‐2.39	.25
Tumor site, ref oral cavity												
Oropharynx	0.32	0.12‐0.83	.02	0.14	0.04‐0.50	.003	0.57	0.37‐0.87	.009	0.90	0.52‐1.56	.70
Larynx	1.75	0.81‐3.79	.16	1.43	0.47‐4.32	.53	1.88	1.09‐3.26	.02	1.95	0.98‐3.85	.06
TNM classification, ref early												
Advanced	0.76	0.39‐1.50	.43	1.31	0.50‐3.40	.58	0.40	0.27‐0.59	< .001	0.49	0.28‐0.86	.01
Comorbidity, ref none or mild												
Moderate or severe	0.87	0.37‐2.06	.76	0.74	0.29‐1.87	.52	0.96	0.58‐1.58	.88	0.85	0.49‐1.47	.57
Treatment received, ref surgery only												
Surgery + adjunct	0.59	0.21‐1.65	.31	0.91	0.29‐2.88	.87	0.46	0.26‐0.79	.005	0.65	0.34‐1.25	.19
Combined chemoradiotherapy	0.74	0.30‐1.82	.51	2.11	0.53‐8.36	.29	0.42	0.25‐0.70	.001	0.67	0.32‐1.39	.28
Radiotherapy only	1.08	0.44‐2.66	.87	0.92	0.28‐3.03	.89	0.96	0.51‐1.78	.89	0.58	0.28‐1.24	.16
BL smoking status, ref never smoked												
Current	2.05	0.57‐7.35	.27	1.78	0.45‐6.96	.41	2.28	1.26‐4.16	.007	2.45	1.23‐4.88	.01
Former	2.95	1.11‐7.86	.03	2.74	0.95‐7.89	.06	1.58	0.94‐2.63	.08	1.51	0.86‐2.66	.16

Abbreviations: BL, baseline; CI, confidence interval; OR, odds ratio; ref, reference.

a Mutually adjusted for all exposures and confounders.

**Table 3:** Unadjusted and mutually adjusted logistic regression models for odds of being a smoker at 12 months postdiagnosis, stratified by prediagnosis smoking status

	Former smoker						Current smoker					
	Unadjusted			Mutually adjusted^a^			Unadjusted			Mutually adjusted^a^		
	OR	95% CI	*P* value	OR	95% CI	*P* value	OR	95% CI	*P* value	OR	95% CI	*P* value
Age, ref < 55 y												
55‐69	0.58	0.25‐1.34	.20	0.52	0.22‐1.28	.16	1.57	0.81‐3.06	.18	1.68	0.78‐3.60	.18
70+	0.47	0.15‐1.46	.19	0.40	0.12‐1.40	.15	1.13	0.42‐3.04	.82	1.30	0.41‐4.09	.66
Sex, ref male												
Female	1.42	0.64‐3.17	.39	1.22	0.81‐2.93	.66	0.79	0.39‐1.59	.51	0.44	0.19‐0.99	.05
Marital status, ref married/cohabiting												
Single, divorced, widowed, or separated	1.64	0.78‐3.47	.19	1.34	0.60‐2.97	.47	1.34	0.77‐2.35	.30	1.14	0.60‐2.17	.68
Education, ref school level												
Further education	0.81	0.37‐1.75	.58	0.77	0.34‐1.71	.52	0.82	0.44‐1.52	.52	0.83	0.40‐1.69	.60
University/poly	0.29	0.07‐1.28	.10	0.27	0.06‐1.26	.10	0.89	0.38‐2.07	.78	0.54	0.21‐1.42	.21
Tumor site, ref oral cavity												
Oropharynx	0.48	0.20‐1.16	.10	0.24	0.07‐0.83	.02	0.85	0.45‐1.61	.62	1.16	0.46‐2.93	.76
Larynx	0.72	0.30‐1.72	.46	0.57	0.18‐1.82	.34	0.24	0.11‐0.56	.001	0.15	0.05‐0.48	.001
TNM classification, ref early												
Advanced	0.97	0.47‐2.00	.94	0.91	0.31‐261	.85	0.65	0.37‐1.14	.14	0.47	0.20‐1.09	.08
Comorbidity, ref none or mild												
Moderate or severe	1.91	0.85‐4.28	.12	1.98	0.82‐4.76	.13	2.84	1.44‐5.60	.003	3.12	1.39‐7.01	.006
Treatment received, ref surgery only												
Surgery + adjunct	0.70	0.22‐2.20	.54	1.17	0.33‐4.21	.81	0.50	0.22‐1.13	.10	0.60	0.22‐1.65	.32
Combined chemoradiotherapy	1.21	0.48‐3.05	.68	3.14	0.74‐13.3	.12	0.72	0.35‐1.46	.35	0.96	0.29‐3.17	.95
Radiotherapy only	0.77	0.26‐2.29	.64	1.23	0.32‐4.71	.76	0.50	0.22‐1.13	.10	1.18	0.37‐3.80	.78
BL alcohol consumption, ref “healthy”												
Unhealthy	0.85	0.41‐1.76	.67	0.86	0.39‐1.88	.70	1.11	0.61‐2.03	.73	0.90	0.45‐1.81	.77

Abbreviations: BL, baseline; CI, confidence interval; OR, odds ratio; ref, reference.

a Mutually adjusted for all exposures and confounders .

In adjusted models, irrespective of their alcohol consumption at diagnosis, women were less likely than men to be high consumers at 12 months (OR 0.48; 95% confidence interval [CI] 0.19‐1.20; *P* = .12 and OR 0.36; 95% CI 0.21‐0.59; *P* < .001 for low and high consumers at diagnosis, respectively; Table 2). Increased alcohol consumption at 12 months was much less likely in people with oropharyngeal cancer compared to oral cavity cancer (OR 0.14; 95% CI 0.04‐0.50; *P* = .003), and more likely in former smokers compared with never smokers (OR 2.74; 95% CI 0.95‐7.89; *P* = .06). Continued high consumption was more likely in people with laryngeal cancer compared to oral cavity cancer (OR 1.95; 95% CI 0.98‐3.85; *P* = .06) and current compared with never smokers (OR 2.45; 95% CI 1.23‐4.88; *P* = .01).

Smoking status at 12 months was only associated with sex and clinical factors. Among former smokers at diagnosis, people with oropharyngeal cancer compared to oral cavity cancer had reduced odds of restarting smoking at 12 months (OR 0.24; 95% CI 0.07‐0.83; *P* = .02; Table 3). Continued smoking at 12 months was less likely in women (OR 0.44; 95% CI 0.19‐0.99; *P* = .05) and people with laryngeal cancer compared to oral cavity cancer (OR 0.15; 95% CI 0.05‐0.48; *P* = .001). Whereas people with moderate or severe comorbidities (OR 3.12; 95% CI 1.39‐7.01; *P* = .006) were more likely to continue smoking.

### 3.4 Logistic regression models for health behaviors at 4 months

These results are described in full in the Supporting Information. In summary, unlike at 12 months, high alcohol consumption was associated with treatment received, specifically having surgery. Continued smoking at 4 months was negatively associated with having advanced‐stage cancer and positively associated with having more severe comorbidities among both former and current smokers. Resumption of smoking was associated with not being in a relationship and with high alcohol consumption at diagnosis.

### 3.5 Sensitivity analyses

Health behaviors at each time point were comparable using time‐point‐specific study samples (Supporting Information Table S4) as when using the main “complete case” study sample (Table 1). Using the previous sex‐specific guidelines for weekly alcohol consumption, the strength of sex associations with alcohol consumption at 4 months and 12 months was comparable for initially low consumers at diagnosis (OR 0.42; 95% CI 0.16‐1.10; *P* = .08 and OR 0.42; 95% CI 0.16‐1.07; *P* = .07) and for initially high consumers at 4 months (OR 0.46; 95% CI 0.27‐0.80; *P* = .006) but did not persist for initially high consumers at 12 months (OR 0.79; 95% CI 0.48‐1.31; *P* = .37).

## 4 DISCUSSION

We analyzed data from 973 people with oral cavity, oropharyngeal, or laryngeal cancer, of whom 54% consumed above the recommended weekly maximum units of alcohol and 21% were current smokers at diagnosis. Twelve months after diagnosis, 60% of people who smoked and 31% of people who consumed alcohol above the recommended weekly limit at baseline had made positive changes to these behaviors. However, these changes were not 1‐way or static over the first year after diagnosis. We found that being male, having a laryngeal tumor, and being a former or current smoker were associated with high alcohol consumption at 12 months. Continuing to smoke at 12 months was associated with being male, having an oral cavity or oropharyngeal tumor, and having more severe comorbidities at diagnosis.

The proportion of current smokers at diagnosis who had quit smoking at 12 months in our study (60%) is comparable to a similar population of people with head and neck cancer (58%),^18^ and higher than in people with other smoking‐related cancers (46%).^34^ The reduction in the prevalence of high alcohol consumption in our study (54% to 41%) was less than the reduction in prevalence of “problem drinking” (25% to 11%) reported by Duffy et al.^18^ Although, our reduction in “harmful” alcohol consumption (17% to 8%) was more comparable, suggesting the threshold for “harmful” consumption in our study may be a more appropriate proxy measure of problem drinking.

The changes in behavior we observed in our study were not stable over the first 12 months after diagnosis. The reduced prevalence of high alcohol consumption from 54% at diagnosis to 41% at 12 months overlooks a larger reduction at 4 months (54% to 35%); it also hides the fact that a large proportion (31%) of high consumers at 12 months had increased their alcohol consumption from 4 months. In comparison, smoking prevalence in this cohort reduced between diagnosis and 4 months and this reduction was maintained at 12 months. Most nonsmokers and former smokers at diagnosis remained nonsmokers but a high proportion of smokers at 12 months (44%) had previously quit smoking either before diagnosis or by 4 months postdiagnosis.

We considered a range of potential factors that might be associated with smoking or alcohol consumption. Few were associated with smoking at 12 months. The reduction in odds of smoking at 12 months among smokers at diagnosis with laryngeal cancer compared to oral cavity cancer is in agreement with previous studies.^32^, ^35^ People who attribute the cause of their head and neck cancer to their own past behavior are more likely to stop smoking,^36^ and smoking is most strongly associated with developing laryngeal cancer compared with other head and neck cancers.^37^ Furthermore, the etiology of head and neck cancer may be changing to include other causal factors, in particular, the emergence of human papillomavirus as a likely cause of oropharyngeal cancer.^38^ As a result, people with laryngeal cancers compared with other head and neck cancers may receive clearer messages about the potential role of smoking in their diagnosis, leading to a reduction in smoking prevalence in this group.

In our study, people with more severe comorbidities had increased odds of resumption or of continued smoking at 4 months, and continued smoking at 12 months. This contrasts with previous studies covering a wider range of cancer diagnoses.^39^, ^40^ We adjusted for severity of comorbidity by using the Adult Comorbidity Evaluation‐27, whereas other studies have adjusted for numbers of comorbidities, which may explain our contrasting results.

The absence of associations in our study between treatment and smoking at 12 months contrasts with previous studies. Functional difficulties after surgery^25^ or the complexity and/or longevity of treatment^32^ were suggested as explanations for lower odds of continued smoking in people receiving surgery with or without adjunct therapy compared to radiotherapy only. Our findings with respect to treatment, therefore, warrant further exploration. Similar to previous studies, treatment was not associated with alcohol consumption at 12 months.^18^, ^23^ We found strong associations between treatment and alcohol consumption at 4 months, with reduced odds of high consumption in people receiving chemotherapy, radiotherapy, or combined therapy rather than surgery alone, irrespective of consumption at diagnosis. This may reflect the continued impact of chemotherapies and radiotherapies on swallowing, taste, or appetite at 4 months, whereas, people who had surgery may have been several weeks into their postoperative recovery period. The absence of associations between treatment and alcohol consumption at 12 months suggests that side effects of chemotherapies and radiotherapies have a transient rather than sustained impact on alcohol consumption.

In our study, the relatively high proportion of people who stopped smoking or reduced their alcohol consumption by 4 months compared with between 4 and 12 months suggests that positive behavior change in this population is most likely in the early months immediately after diagnosis. This compares favorably with the optimal time frame for a smoking cessation intervention in people newly diagnosed with head and cancer.^25^ It is also a similar time frame in which treatment side effects might be affecting smoking behavior, however, the absence of associations between treatment and change in smoking at 4 months in our study does not support this being an important factor. For some people in our study, health behaviors that worsened or improvements were not sustained over the 12 months. Interventions may, therefore, be more successful if they also support the maintenance of behavior change in the 12 months after diagnosis.

We found strong associations between being men and high alcohol consumption, which persisted when using previous sex‐specific thresholds for alcohol consumption. This contrasts with previous studies, which found no association of sex with continuing to consume any alcohol^23^ or with problem drinking behavior.^18^ The authors included alcohol abuse status in their analysis, which has separately been found to be more prevalent in men at the time of diagnosis of head and neck cancer.^18^ It may, therefore, be that the sex association we found is partially or fully explained by a higher prevalence of problem drinking behavior in men in our cohort.

People who continue to smoke and/or consume higher amounts of alcohol after diagnosis have a worse prognosis.^9^, ^10^, ^41^ We found an association between being a current or former smoker at diagnosis and high alcohol consumption at 12 months, in agreement with a previous study.^18^ This group of people who both drink and smoke are less likely to change these behaviors but may be likely to benefit the most from effective interventions that target both behaviors because they have a multiplicative effect compared with targeting each behavior individually.^42^

Compared with previous studies in this area, our study has 2 important strengths. We used data from a large prospective clinical cohort with measures of smoking and alcohol consumption at 3 time points during the first 12 months after diagnosis. The size of our cohort allowed us to stratify our regression models by health behaviors at diagnosis, because reducing unhealthy behaviors is a different outcome from maintaining healthy behaviors. There are several weaknesses to our study. We relied on self‐reported measures for our demographic and health behavior measures. Our measures of smoking and alcohol behavior in particular may be affected by recall and self‐reported biases. For smoking, this may have led to an underestimate of the prevalence of smoking,^43^ whereas these biases seem to be less of a concern for alcohol consumption.^44^ Because we did not study change in the volume of alcohol consumed directly but change in categorized consumption, our analyses should be less sensitive to minor differences due to recall bias. These biases could be further reduced by using objective biomarkers rather than self‐reported measures. Cotinine levels are an accepted indicator of smoking status^43^ but currently there is not a suitable biomarker of alcohol consumption except for acute consumption. Furthermore, we were only able to focus on consumption rather than measures of lifetime exposure to alcohol or tobacco smoke, which was either not collected (alcohol) or had a large proportion of missing data (smoking). The behavior data collected at 4 months are likely to be from a mixture of people who have completed their treatment alongside those still in active treatment, which may mask behavior change and make it harder to interpret because people are at different stages of their treatment. We also had a high proportion of missing data, which may have introduced bias but we did explore the potential impact of this through sensitivity analyses.

Further studies in this population should evaluate whether these short‐term changes in smoking and alcohol consumption persist beyond 1 year and the prognostic implications of these changes. Given the emergence of human papillomavirus as a significant prognostic marker, it might also be of interest to explore whether the benefits of health behavior changes persist within human papillomavirus‐positive head and neck cancers.

## 5 CONCLUSIONS

The 12‐month period immediately after a cancer diagnosis is characterized by important reductions in alcohol consumption and smoking prevalence. However, the majority of high alcohol consumers at diagnosis do not reduce their consumption, and may benefit from assistance both to reduce their consumption and maintain this reduction. Most current smokers at diagnosis stop smoking by 12 months, but former smokers are also at risk of restarting. Furthermore, people who continue smoking 4 months after diagnosis are likely to continue up to 12 months.

## Supporting information

Additional Supporting Information may be found online in the supporting information tab for this article.

Supporting Information
