# Supplementary material for: Change in alcohol and tobacco consumption after a diagnosis of head and neck cancer: Findings from Head and Neck 5000
Source: Head Neck. 2018 Feb 27;40(7):1389–99. doi: 10.1002/hed.25116 (PMC6175475; doi:10.1002/hed.25116)
Supplement: Supplementary file 1 — Supporting Information [file HED-40-1389-s001.docx]

## Supplementary material

### Results

#### Logistic regression models for health behaviours at 4-months

##### Adjusted models

Similar associations were found with health behaviours at 4-months as were found at 12-months. Women were less likely than men to be high consumers of alcohol irrespective of their pre-diagnosis consumption (Table S3). People who were initially high consumers and were diagnosed at an advanced stage were less likely to be high consumers at 4-months. The associations between smoking at diagnosis and 4-month behaviour were generally consistent with the associations with 12-month behaviour. People who were initially high consumers were more likely to remain high consumers if they were a current or former smoker at diagnosis compared with never smokers. People with laryngeal tumours were more likely to be high consumers at 4-months compared with people with oral cavity tumours, irrespective of their consumption at diagnosis (OR=3.02, P=0.05 and OR=1.96, P=0.04 for low and high consumers at diagnosis respectively). Treatment received was associated with alcohol consumption at 4-months. Specifically, people who received chemo-radiotherapy or radiotherapy were less likely to be high consumers at 4-months compared with people who had surgery only, irrespective of consumption at diagnosis. High consumers at diagnosis who had surgery and adjunct therapy were also less likely to continue to be high consumers at 4-months than people who had surgery only.

For smoking, unlike at 12-months, some factors were associated with resumption of smoking among former smokers (Table S4). Compared with people who were married or living with a partner, people who were single, divorced, widowed or separated were more likely to restart smoking (OR=2.35, P=0.06). But there was no association between marital status and continued smoking at 4-months. There was weak evidence that older people were more likely than younger people to continue smoking (OR=1.85, P=0.12 and OR=2.66, P=0.09 for 55-69 and 70+ years old respectively). Being diagnosed with HNC at an advanced stage was associated with reduced odds of continuing smoking at 4-months. People with moderate or severe co-morbidities at diagnosis were more likely to restart (OR=2.73, P=0.04) or continue smoking (OR=3.66, P=0.001) at 4-months. High alcohol consumption at diagnosis was strongly associated with restarting smoking (OR=3.39, P=0.03) at 4-months, but not with continuing smoking.

| **Table S1: A comparison of people eligible to participate with and without complete health behaviour data at diagnosis, 4-months and 12-months** | | | |
| --- | --- | --- | --- |
| **Factor** | **Incomplete data**  **Number of people (%)** | **Complete data**  **Number of people (%)** | **p-value** |
| **Number of people** | 1945 | 973 |  |
| **Baseline - Age at date of consent** (years), mean (SD) | 61.0 (11.2) | 61.8 (10.1) | 0.05 |
| **Baseline – Sex** |  |  | 0.04 |
| Male | 1417 (72.9%) | 743 (76.4%) |  |
| Female | 528 (27.1%) | 230 (23.6%) |  |
| **Marital status** |  |  | 0.98 |
| Married/cohabiting | 1314 (68.8%) | 669 (68.8%) |  |
| Single, divorced, widowed or separated | 596 (31.2%) | 304 (31.2%) |  |
| **Education** |  |  | 0.08 |
| School level | 865 (48.6%) | 437 (44.9%) |  |
| Further Education | 613 (34.4%) | 341 (35.0%) |  |
| University/Poly | 302 (17.0%) | 195 (20.0%) |  |
| **Tumour site** |  |  | 0.31 |
| Oral Cavity | 568 (29.2%) | 308 (31.7%) |  |
| Oropharynx | 894 (46.0%) | 421 (43.3%) |  |
| Larynx | 483 (24.8%) | 244 (25.1%) |  |
| **Baseline TNM stage** |  |  | 0.06 |
| Early stage | 815 (42.0%) | 444 (45.6%) |  |
| Advanced stage | 1124 (58.0%) | 529 (54.4%) |  |
| **Baseline co-morbidity** |  |  | 0.61 |
| No co-morbidity, mild decompensation or unknown | 1572 (80.8%) | 794 (81.6%) |  |
| Moderate or severe decompensation | 373 (19.2%) | 179 (18.4%) |  |
| **Treatment received** |  |  | 0.40 |
| Surgery only | 473 (24.3%) | 254 (26.1%) |  |
| Surgery + adjunct | 497 (25.6%) | 222 (22.8%) |  |
| Combined chemoradiotherapy | 591 (30.4%) | 302 (31.0%) |  |
| Radiotherapy only | 382 (19.7%) | 195 (20.0%) |  |
| **BL - Grouped weekly alcohol consumption** |  |  | 0.002 |
| Non-drinker | 509 (27.5%) | 242 (24.9%) |  |
| Moderate | 454 (24.5%) | 203 (20.9%) |  |
| Hazardous | 668 (36.0%) | 367 (37.7%) |  |
| Harmful | 223 (12.0%) | 161 (16.5%) |  |
| **4M - Grouped weekly alcohol consumption** |  |  | <0.001 |
| Non-drinker | 533 (44.3%) | 415 (42.7%) |  |
| Moderate | 330 (27.5%) | 216 (22.2%) |  |
| Hazardous | 286 (23.8%) | 272 (28.0%) |  |
| Harmful | 53 (4.4%) | 70 (7.2%) |  |
| **12M - Grouped weekly alcohol consumption** |  |  | <0.001 |
| Non-drinker | 386 (38.2%) | 320 (32.9%) |  |
| Moderate | 303 (30.0%) | 250 (25.7%) |  |
| Hazardous | 273 (27.0%) | 327 (33.6%) |  |
| Harmful | 49 (4.8%) | 76 (7.8%) |  |
| **BL - Tobacco** |  |  | 0.05 |
| Current user | 326 (18.1%) | 204 (21.0%) |  |
| Former | 1030 (57.3%) | 563 (57.9%) |  |
| Never | 442 (24.6%) | 206 (21.2%) |  |
| **4M - Tobacco** |  |  | 0.29 |
| Current User | 91 (12.4%) | 105 (10.8%) |  |
| Former or Never | 640 (87.6%) | 868 (89.2%) |  |
| **12M - Tobacco** |  |  | 0.63 |
| Current User | 70 (10.9%) | 114 (11.7%) |  |
| Former or Never | 570 (89.1%) | 859 (88.3%) |  |

| **Table S2: Unadjusted and adjusted logistic regression models for odds of consuming alcohol above the recommended limits at 4-months post-diagnosis, stratified by pre-diagnosis alcohol consumption** | | | | | | | | | | | | |
| --- | --- | --- | --- | --- | --- | --- | --- | --- | --- | --- | --- | --- |
|  | **Low Consumer** | | | | | | **High Consumer** | | | | | |
|  | **Unadjusted** | | | **Mutually adjusted^*^** | | | **Unadjusted** | | | **Mutually adjusted^*^** | | |
|  | **OR** | ***95% CI*** | **P** | **OR** | ***95% CI*** | **P** | **OR** | ***95% CI*** | **P** | **OR** | ***95% CI*** | **P** |
| **Age** (ref <55 years) |  |  |  |  |  |  |  |  |  |  |  |  |
| 55-69 | 0.80 | *0.34-1.89* | 0.61 | 0.73 | *0.29-1.86* | 0.51 | 1.50 | *0.97-2.31* | 0.07 | 1.44 | *0.88-2.36* | 0.14 |
| 70+ | 1.29 | *0.53-3.14* | 0.58 | 0.97 | *0.35-2.69* | 0.95 | 1.45 | *0.81-2.60* | 0.21 | 1.12 | *0.57-2.20* | 0.75 |
| **Gender** (ref Male) |  |  |  |  |  |  |  |  |  |  |  |  |
| Female | 0.38 | *0.15-0.93* | 0.03 | 0.35 | *0.13-0.92* | 0.03 | 0.41 | *0.26-0.66* | <0.001 | 0.30 | *0.17-0.51* | <0.001 |
| **Marital status** (ref married/cohabiting) |  |  |  |  |  |  |  |  |  |  |  |  |
| Single, divorced, widowed or separated | 0.82 | *0.39-1.74* | 0.61 | 0.81 | *0.35-1.85* | 0.62 | 0.95 | *0.65-1.37* | 0.78 | 0.77 | *0.50-1.19* | 0.24 |
| **Education** (ref School level) |  |  |  |  |  |  |  |  |  |  |  |  |
| Further Education | 0.97 | *0.46-2.03* | 0.94 | 0.87 | *0.39-1.93* | 0.73 | 1.24 | *0.84-1.84* | 0.27 | 1.33 | *0.86-2.07* | 0.20 |
| University/Poly | 0.60 | *0.22-1.65* | 0.32 | 0.59 | *0.19-1.81* | 0.36 | 0.86 | *0.54-1.37* | 0.52 | 1.11 | *0.65-1.89* | 0.71 |
| **Tumour site** (ref Oral cavity) |  |  |  |  |  |  |  |  |  |  |  |  |
| Oropharynx | 0.51 | *0.21-1.21* | 0.13 | 0.83 | *0.28-2.48* | 0.74 | 0.55 | *0.37-0.82* | 0.004 | 1.05 | *0.61-1.84* | 0.85 |
| Larynx | 1.65 | *0.74-3.69* | 0.23 | 3.02 | *1.02-8.95* | 0.05 | 2.07 | *1.27-3.38* | 0.004 | 1.96 | *1.03-3.76* | 0.04 |
| **TNM stage** (ref Early) |  |  |  |  |  |  |  |  |  |  |  |  |
| Advanced | 0.86 | *0.44-1.68* | 0.66 | 1.41 | *0.58-3.39* | 0.45 | 0.29 | *0.20-0.42* | <0.001 | 0.39 | *0.23-0.66* | <0.001 |
| **Co-morbidity** (ref None or mild) |  |  |  |  |  |  |  |  |  |  |  |  |
| Moderate or severe | 1.24 | *0.56-2.73* | 0.59 | 1.19 | *0.49-2.85* | 0.70 | 0.96 | *0.60-1.53* | 0.87 | 0.87 | *0.51-1.49* | 0.61 |
| **Treatment received** (ref surgery only) |  |  |  |  |  |  |  |  |  |  |  |  |
| Surgery + adjunct | 0.86 | *0.37-2.00* | 0.73 | 0.77 | *0.28-2.13* | 0.61 | 0.21 | *0.12-0.36* | <0.001 | 0.26 | *0.14-0.49* | <0.001 |
| Combined chemoradiotherapy | 0.27 | *0.09-0.78* | 0.02 | 0.21 | *0.05-0.83* | 0.03 | 0.29 | *0.18-0.47* | <0.001 | 0.43 | *0.21-0.87* | 0.02 |
| Radiotherapy only | 0.56 | *0.21-1.44* | 0.23 | 0.21 | *0.06-0.69* | 0.01 | 0.78 | *0.44-1.39* | 0.40 | 0.42 | *0.20-0.87* | 0.02 |
| **BL smoking status** (ref Never smoked) |  |  |  |  |  |  |  |  |  |  |  |  |
| Current | 2.78 | *0.98-7.84* | 0.05 | 2.14 | *0.69-6.66* | 0.19 | 2.08 | *1.18-3.67* | 0.01 | 1.96 | *1.00-3.83* | 0.05 |
| Former | 1.57 | *0.65-3.80* | 0.32 | 1.12 | *0.42-2.99* | 0.83 | 2.19 | *1.32-3.64* | 0.002 | 1.99 | *1.12-3.54* | 0.02 |

* Mutually adjusted for all exposures and confounders

| **Table S3: Unadjusted and adjusted logistic regression models for odds of being a smoker at 4-months post-diagnosis, stratified by pre-diagnosis smoking status** | | | | | | | | | | | | |
| --- | --- | --- | --- | --- | --- | --- | --- | --- | --- | --- | --- | --- |
|  | **Former Smoker** | | | | | | **Current Smoker** | | | | | |
|  | **Unadjusted** | | | **Mutually adjusted^*^** | | | **Unadjusted** | | | **Mutually adjusted^*^** | | |
|  | **OR** | ***95% CI*** | **P** | **OR** | ***95% CI*** | **P** | **OR** | ***95% CI*** | **P** | **OR** | ***95% CI*** | **P** |
| **Age** (ref <55 years) |  |  |  |  |  |  |  |  |  |  |  |  |
| 55-69 | 1.37 | *0.45-4.16* | 0.58 | 1.13 | *0.35-3.66* | 0.84 | 1.79 | *0.90-3.59* | 0.10 | 1.85 | *0.85-4.00* | 0.12 |
| 70+ | 0.66 | *0.14-3.02* | 0.59 | 0.54 | *0.10-2.81* | 0.47 | 2.4 | *0.90-6.43* | 0.08 | 2.66 | *0.86-8.19* | 0.09 |
| **Gender** (ref Male) |  |  |  |  |  |  |  |  |  |  |  |  |
| Female | 1.14 | *0.44-2.94* | 0.78 | 1.22 | *0.43-3.47* | 0.71 | 0.87 | *0.43-1.77* | 0.71 | 0.61 | *0.27-1.38* | 0.23 |
| **Marital status** (ref married/cohabiting) |  |  |  |  |  |  |  |  |  |  |  |  |
| Single, divorced, widowed or separated | 2.22 | *0.97-5.07* | 0.06 | 2.35 | *0.96-5.77* | 0.06 | 1.34 | *0.76-2.36* | 0.31 | 1.29 | *0.68-2.46* | 0.43 |
| **Education** (ref School level) |  |  |  |  |  |  |  |  |  |  |  |  |
| Further Education | 1.12 | *0.47-264* | 0.80 | 1.07 | *0.43-265* | 0.88 | 0.65 | *0.34-1.23* | 0.19 | 0.69 | *0.34-1.41* | 0.31 |
| University/Poly | 0.45 | *0.10-2.04* | 0.30 | 0.47 | *0.10-2.24* | 0.34 | 1.07 | *0.46-2.47* | 0.88 | 0.89 | *0.34-2.30* | 0.81 |
| **Tumour site** (ref Oral cavity) |  |  |  |  |  |  |  |  |  |  |  |  |
| Oropharynx | 0.48 | *0.16-1.42* | 0.19 | 0.39 | *0.10-1.56* | 0.19 | 0.75 | *0.39-1.44* | 0.39 | 0.92 | *0.36-2.34* | 0.86 |
| Larynx | 1.11 | *0.43-2.90* | 0.83 | 1.37 | *0.40-4.75* | 0.62 | 0.56 | *0.27-1.18* | 0.13 | 0.52 | *0.19-1.47* | 0.22 |
| **TNM stage** (ref Early) |  |  |  |  |  |  |  |  |  |  |  |  |
| Advanced | 1.29 | *0.56-2.96* | 0.55 | 2.52 | *0.76-8.35* | 0.13 | 0.60 | *0.34-1.06* | 0.08 | 0.43 | *0.19-0.99* | 0.05 |
| **Co-morbidity** (ref None or mild) |  |  |  |  |  |  |  |  |  |  |  |  |
| Moderate or severe | 2.34 | *0.97-5.63* | 0.06 | 2.733 | *1.04-7.17* | 0.04 | 4.07 | *2.03-8.17* | <0.001 | 3.66 | *1.67-8.02* | 0.001 |
| **Treatment received** (ref surgery only) |  |  |  |  |  |  |  |  |  |  |  |  |
| Surgery + adjunct | 0.48 | *0.12-1.88* | 0.29 | 0.54 | *0.11-2.58* | 0.44 | 0.44 | *0.19-1.04* | 0.06 | 0.53 | *0.19-1.47* | 0.22 |
| Combined chemoradiotherapy | 1.03 | *0.37-2.83* | 0.96 | 0.99 | *0.22-4.47* | 0.99 | 0.76 | *0.37-1.55* | 0.45 | 1.12 | *0.35-3.63* | 0.85 |
| Radiotherapy only | 0.74 | *0.23-2.38* | 0.61 | 0.88 | *0.22-3.52* | 0.86 | 0.63 | *0.28-1.43* | 0.27 | 0.63 | *0.21-1.90* | 0.42 |
| **BL alcohol consumption** (ref ‘healthy’) |  |  |  |  |  |  |  |  |  |  |  |  |
| Unhealthy | 3.20 | *1.18-8.70* | 0.02 | 3.39 | *1.16-9.91* | 0.03 | 0.90 | *0.49-1.64* | 0.72 | 0.76 | *0.38-1.51* | 0.43 |

* Mutually adjusted for all exposures and confounders

| **Table S4: Categories of alcohol and tobacco consumption at diagnosis, 4-months and 12-months** | | | |
| --- | --- | --- | --- |
|  | **Diagnosis** | **4 months** | **12 months** |
| **Grouped weekly alcohol consumption** | **BL sample^**  **N=2,703** | **4M sample^**  **N=1,676** | **12M sample^**  **N=1,588** |
| ‘Healthy’ drinker (≤14 units/week) | 1,331 (49.2%) | 1,129 (67.4%) | 982 (61.8%) |
| Non-drinker | 707 (26.2%) | 719 (42.9%) | 557 (35.1%) |
| Moderate use | 624 (23.1%) | 410 (24.5%) | 425 (26.8%) |
| ‘Unhealthy’ drinker (>14 units/week) | 1,372 (50.8%) | 547 (32.6%) | 606 (38.2%) |
| Hazardous use | 999 (37.0%) | 441 (26.3%) | 498 (31.4%) |
| Harmful use | 373 (13.8%) | 106 (6.3%) | 108 (6.8%) |
| **Tobacco consumption** |  |  |  |
| Current smoker | 517 (19.1%) | 191 (11.4%) | 182 (11.5%) |
| Former* | 1,550 (57.3%) | 1,485 (88.6%) | 1,406 (88.5%) |
| Never* | 636 (23.5%) |  |  |

* - ‘Former’ and ‘Never’ are combined at the 4-month and 12-month questionnaires

^ - Study samples defined for the diagnosis, 4-month and 12-month time-point separately
